# Membrane-bound IL-6R is upregulated on Th17 cells and inhibits Treg cell migration by regulating post-translational modification of VASP in autoimmune arthritis

**DOI:** 10.1007/s00018-021-04076-2

**Published:** 2021-12-16

**Authors:** Shuaifeng Yan, Viktoria Golumba-Nagy, Konstantin Kotschenreuther, Jan Thiele, Nasrin Refaian, Deng Shuya, Lydia Gloyer, Mara Dittrich-Salamon, Anja Meyer, Ludwig M. Heindl, David M. Kofler

**Affiliations:** 1 Laboratory of Molecular Immunology, Division of Rheumatology and Clinical Immunology, Department I of Internal Medicine, University of Cologne, Kerpenerstr. 62, 50937 Cologne, Germany; 2 Department of Ophthalmology, University of Cologne, Cologne, Germany; 3 Center for Integrated Oncology, Aachen Bonn Cologne Duesseldorf, Cologne, Germany

**Keywords:** Regulatory T cells, Autoimmune arthritis, IL-6, T cell migration, VASP, Collagen-induced arthritis

## Abstract

Autoimmune arthritis is characterized by impaired regulatory T (Treg) cell migration into inflamed joint tissue and by dysregulation of the balance between Treg cells and Th17 cells. Interleukin-6 (IL-6) is known to contribute to this dysregulation, but the molecular mechanisms behind impaired Treg cell migration remain largely unknown. In this study, we assessed dynamic changes in membrane-bound IL-6 receptor (IL6R) expression levels on Th17 cells by flow cytometry during the development of collagen-induced arthritis (CIA). In a next step, bioinformatics analysis based on proteomics was performed to evaluate potential pathways affected by altered IL-6R signaling in autoimmune arthritis. Our analysis shows that membrane-bound IL-6R is upregulated on Th17 cells and is inversely correlated with IL-6 serum levels in experimental autoimmune arthritis. Moreover, IL-6R expression is significantly increased on Th17 cells from untreated patients with rheumatoid arthritis (RA). Interestingly, CD4^+^ T cells from CIA mice and RA patients show reduced phosphorylation of vasodilator-stimulated phosphoprotein (VASP). Bioinformatics analysis based on proteomics of CD4^+^ T cells with low or high phosphorylation levels of VASP revealed that integrin signaling and related pathways are significantly enriched in cells with low phosphorylation of VASP. Specific inhibition of p-VASP reduces the migratory function of Treg cells but has no influence on effector CD4^+^ T cells. Importantly, IL-6R blockade restores the phosphorylation level of VASP, thereby improving the migratory function of Treg cells from RA patients. Thus, our results establish a link between IL6R signaling and phosphorylation of VASP, which controls Treg cell migration in autoimmune arthritis.

**Supplementary Information:**

The online version contains supplementary material available at 10.1007/s00018-021-04076-2.

## Introduction

Rheumatoid arthritis (RA) is one of the most prevalent autoimmunity diseases and is characterized by chronic inflammation, bone destruction and extra-articular manifestations [1–4]. Although the pathogenesis of RA is not fully understood, evidence supports the concept that an imbalance between regulatory T (Treg) and Th17 cell subsets play a major role in the pathogenesis of RA [5, 6]. However, the pathogenic mechanism leading to dysregulation of Treg cells and Th17 cells in RA remains unclear. CD4^+^ T cell subsets cells are differentiated from naive CD4^+^ T cells in the presence of specific cytokines and are characterized by specific biological functions and gene expression profiles [4]. The frequency of Th17 cells and the level of IL-17 are increased in the peripheral blood and in the synovial tissue of RA patients as compared to healthy control. Interestingly, Th17 cell frequencies correlate with disease activity [7, 8]. Moreover, Th17 cells have been reported to recruit neutrophils, activate B cells and to promote osteoclastogenesis [9, 10]. Treg cells can specifically suppress the activity of Th17 cells and other effector T cells, thereby maintaining self-tolerance and preventing autoimmunity. Treg cells act through direct cell contact or by producing soluble molecules including IL-10 and transforming growth factor β (TGF- β). Many studies observed reduced circulating Treg cell numbers in RA as compared to healthy individuals [11, 12]. In contrast, some researchers report a higher level of Treg cells in the synovial fluid [13, 14]. This contradictory results may be explained by different strategies used to identify Treg cells and by inhomogeneity of patient populations. Moreover, it has been shown that the suppressive capacity of Treg cells gets inhibited in the synovial fluid of RA patients while Treg cells in peripheral blood remain suppressive [15]. Importantly, Treg cells can differentiate into Th17 cells and get accumulated at inflammatory area [9, 10]. Due to the complexity and interactions between Treg and Th17 subsets, the underlying molecular mechanism regulating their interaction needs to be investigated to better understand their role in RA pathogenesis.

IL-6 plays a crucial role in RA pathogenesis and drives Th17 cell differentiation together with IL-1β, TGF-β and IL-23 [16, 17]. Recently, Harbour et al*.* reported that classical IL-6 signaling through continued membrane-bound IL-6 receptor is required for both, the development of Th17 cells and for the retainment of the transcriptional and functional characteristics of Th17 cells [18]. It has also been shown that the level of vasodilator-stimulated phosphoprotein (VASP) in endothelial HMEC-1 cells is reduced following in vitro cultivation in the presence of IL-6 [19]. This observation is important as VASP is a major regulator of cell migration in fibroblasts and cancer cells, thereby linking IL-6 signaling to cell migration [20–23]. Although the role of IL-6 signaling in Th17 cell induction is well established [24, 25], it remains unclear how variation of membrane-bound IL-6 receptor expression on Th17 cells affects the interaction between Th17 cells and Treg cells during the development of autoimmune arthritis. This study aimed to reveal potential dynamic changes in membrane-bound IL-6 receptor expression as well as possible effects of altered classical IL-6 receptor signaling on Th17 and Treg cell functions.

## Materials and methods

### Patients

A total of 33 healthy individuals and 65 RA patients were enrolled in this study. All RA patients fulfilled the criteria of the 2010 ACR/ EULAR classification [26]. Peripheral blood of patients and healthy individuals were collected at the outpatient clinic at the University Hospital Cologne. The patients’ characteristics are provided in Table 1. Untreated RA patients were defined as either first diagnosed or patients without treatment for at least 8 weeks before inclusion in the study. Age and sex-matched healthy individuals served as controls. Blood was drawn after written informed consent was obtained in accordance with the Declaration of Helsinki. The study was approved by the Ethics Committee of the University Hospital Cologne (approval no. 13-091).

**Table 1:** Patients’ characteristics of RA patients and healthy individuals

Samples	Nr	Sex	Age	Duration (years)	DAS28	RF	ACPA	CRP	ESR
HC	33	69.7% F	56.18 ± 3.83	n/a	0	neg	neg	n/a	n/a
RA	65	66.2% F	56.15 ± 3.85	4.23 ± 0.75	3.76 ± 0.37	188.22 ± 64.01	391.08 ± 64.01	21.08 ± 5.84	27.42 ± 5.89

Blood samples were collected from patients with rheumatoid arthritis (RA) and healthy individuals. RA untreated patients were defined as either newly diagnosed with RA or untreated for at least 8 weeks. *neg*. negative; *n/a* not applicable

### Mice and collagen type II induce arthritis

Mice of the DBA/1 J strain (from Jackson lab, *n* = 117) were used for the induction of collagen type II induced arthritis (CIA). Experiments involving the CIA mouse model were performed as described previously by Brand et al.[27]. Briefly, all mice were kept under specific pathogen-free conditions on a 12 h reversed light/dark cycle, 8–12 weeks old mice were matched by age and sex in all experiments unless specifically stated. The local authorities and animal protection committee approved animal experiments in this study (LANUV NRW, approval no. 81-02.04.2018 A161). Emulsion of bovine type II collagen (Chondrex Inc, Woodinville, USA) with complete Freund’s adjuvant (CFA) containing 0.5 mg/ml of inactivated mycobacterium tuberculosis (Chondrex Inc, Woodinville, USA) was prepared by the Ika T8 Ultra Turrax homogenizer (IKA-Werke GmbH & Co, Baden-Württemberg, Germany). Then the stability of the emulsion was tested by adding one drop of the emulsion into a beaker of water. The emulsion was kept on ice and quickly transferred to an animal facility for the injection immunization. The mice were injected intradermally at the base of the tail with a total emulsion volume of 100 μl at the beginning of the induction and a booster injection of 100 μl of CII emulsified with incomplete Freund’s adjuvant (IFA) (Chondrex Inc, Woodinville, USA) was performed in CIA group at Day 21 while the mice in the control group were injected with same volume Dulbecco’s phosphate buffered saline (Gibco^®^ DPBS, New York, USA). The mouse paw thickness and clinical score were collected once a week by blinded independent people to their treatment for the first 3 weeks and every three days for the next 5 weeks. The clinical score of arthritis was graded on a scale of 0–4 scales as follows: grade 0, no swelling and no erythema; grade 1, slight swelling and erythema; grade 2, moderate swelling and edema; grade 3, severe swelling and pronounced edema; and grade 4, severe swelling and edema with joint rigidity as previously described [28]. The maximum score is 16 for each mouse, and each limb was scored independently.

### T cell isolation

Primary human peripheral blood monocyte cells (PBMCs) was isolated by density gradient centrifugation (PAN™-Biotech GmbH, Aidenbach, Germany). We purified CD4^+^ T cells by magnetic-activated cell sorting (MACS) based on the negative selection with a human CD4^+^ T cell isolation kit (Miltenyi Biotec, Bergisch Gladbach, Germany). Naïve CD4^+^ T cells were magnetically isolated with the Naive T Cell Isolation Kit for human (Miltenyi Biotec, Bergisch Gladbach, Germany). The purity of the isolated cell populations was verified by flow cytometry and only the samples with a purity of more than 95% were used for subsequent experiments. Viable cells were counted using the automated cell counter CellCountess (Life Technologies GmbH, Darmstadt, Germany).

### Th17 cell subset induction

Naïve CD4^+^ T cells were cultured in X-Vivo 15 media (Lonza, Cologne, Germany), which combines with 1% human serum and 1% penicillin–streptomycin (Both from Sigma-Aldrich, Saint Louis, USA). For the activation of the T cell receptor (TCR), cells were incubated with the antibodies from T cell Activation/Expansion Kit (Miltenyi Biotec, Bergisch Gladbach, Germany). For the induction of Th17 cells, recombinant human IL-1β (12.5 ng/μl), IL-6 (25 ng/ml; Both from Miltenyi Biotec, Bergisch Gladbach, Germany), recombinant human IL-23 (25 ng/ml; PeproTech, Rocky Hill, USA), and recombinant human TGF-β (25 ng/μl; PAN™-Biotech GmbH, Aidenbach, Germany) were added to cell culture for 96 h as described previously [29]. To ensure a high induction quality, we changed the medium and cytokines after 72 h of incubation. The induced Th17 cells were collected for quantitative real-time PCR.

### Flow cytometry

Purified CD4^+^ T cells or induced Th17 cells were stimulated for six hours with PMA (500 ng/ml; Abcam, Cambridge, UK) and ionomycin (1.5 μM; Cell Signaling Technology^®^, Danvers, USA). Brefeldin A (eBioscience, San Diego, USA) was added to cell culture two hours before flow cytometry staining. Dead cells were excluded by the LIVE/DEAD™ Fixable Dead Cell Stain Kit (Invitrogen, Thermo Fisher Scientific, Carlsbad, USA). Briefly, cells were stained with cell surface targets including CD4, CD25, and CD127, then fixed and permeabilized by the BD Cytofix/Cytoperm Kit (BD Bioscience, Heidelberg, Germany) or the True-Nuclear™ Transcription Factor Buffer Set (BioLegend Inc., San Diego, USA) according to the manufacturer’s instructions and stained with anti-IL17A, anti-IFN-γ, and anti-FoxP3 (all antibodies and isotype antibodies from Bio Legend Inc., San Diego, USA). Th17 cells were identified by IL-17A expression in purified CD4^+^ T cells. Treg cells were defined as CD4^+^CD25^+^CD127^−^FoxP3^+^ T cells in humans and CD4^+^FoxP3^+^ T cells in mice. Flow cytometry was performed on the Gallios 10/3 flow cytometer and the results were analyzed by Kaluza Analysis Software (Both from Beckman Coulter, Krefeld, Germany).

### *Mouse CD4*^+^*T cells isolation*

At the end of the experiment, mice were anesthetized using 4% isoflurane in the air for blood collection and subsequent organs harvest to collect blood serum and splenic lymphocytes. Mouse spleens were pooled with 70 μm strainer (Corning™ Incorporated Costar, New York, USA) and 1 ml Syringe handle (B. Braun Melsungen AG, Melsungen, Germany) to get single-cell suspensions, and the suspensions were filtered twice with strainers. Then blood cells from the splenic suspensions got lysed with red blood cell lysing buffer (Bio Legend Inc., San Diego, USA). Density gradient centrifugation (PAN™-Biotech GmbH, Aidenbach, Germany) was used to isolate primary mouse monocytes from single-cell suspensions. Mouse CD4^+^ T cells were enriched from mice splenic single-cell suspension using the CD4^+^ T cell isolation kit for mouse (Miltenyi Biotec, Bergisch Gladbach, Germany). The purity of the isolated cell was verified by flow cytometry, and only the samples with a purity of more than 95% were used for subsequent experiments.

### Quantitative real-time PCR

Primers for IL-6 receptor and β2-microglobulin were purchased from Applied Biosystems. All reactions were performed using the 7500 Fast Real-Time PCR System (Applied Biosystems). The values are represented as the difference in Ct values normalized to β2-microglobulin for each sample using the following formula: relative RNA expression = (2-dCt) × 10^3^. The primer sequences for human primers were listed as follows: IL-6 receptor FW: CGTCAGCTCCACATCTGATAGTG, RV: CCTTTGGAGCCCCTTTCTG; β2-microglobulin FW: TGTCCACCTTCCAGCAGATGT, RV: AGCTCAGTA ACAGTCCGCCTAG.

### H&E staining

For histological analysis, limbs were collected after the mice were sacrificed at the end of the experiment and fixed in 10% neutral buffered formalin for 24 h at 4 degree and decalcified in 20% EDTA (Both from Sigma-Aldrich, Saint Louis, USA) solution on shaking bed at 4 degree for 4–6 weeks. Then the limbs were embedded in paraffin. Sections were cut as thick as 5 μm. Then sections were deparaffinized in xylene, dehydrated with graded ethanol and stained with hematoxylin and eosin (H&E staining). Histopathological scores were separately scored by two independent researchers in a blinded manner as described previously [30].

### Immunohistochemistry staining

For immunohistochemistry staining, tissue peroxidase was blocked with 3.0% hydrogen peroxide in methanol for 20 min at room temperature. For antigen retrieval, the citric acid buffer was used and the slides were heated at 100 °C for 20 min and then cooled for 30 min at room temperature. After washing, the sections were incubated with goat serum (Abcam, Cambridge, UK) to reduce unspecific protein binding after deparaffinization and dehydration. IL17 expression was detected with a polyclonal rabbit anti-IL17 antibody (Abcam, Cambridge, UK). After washing, Rabbit AP Polymer (Abcam, Cambridge, UK) was added to each section for 30 min in a moist chamber. Apply Permanent Red Working Solution (Abcam, Cambridge, UK) to completely cover the tissue for 10 min. We then counterstained with Meyer’s hematoxylin. As a positive control, human tonsil tissue was used.

### Western blot analysis

Purified human and murine CD4^+^ cells were lysed with cell lysis buffer (BioLegend Inc., San Diego, USA), and protein concentration was detected with the BCA Protein Assay Kit (Cell Signaling Technology^®^, Danvers, USA). Lysates were run on 4–15% gradient polyacrylamide gels (Bio-Rad Laboratories, Munich, Germany). Blotting was performed with the TransBlot^®^ Turbo™ Transfer System (Bio-Rad Laboratories). Proteins were detected with the following antibodies: HRP anti-β2-Actin antibody mouse mAb (Abcam, Cambridge, UK), anti-VASP rabbit mAb, and anti-rabbit IgG HRP-linked antibody (Both from Cell Signaling Technology^®^, Danvers, USA). Detection was performed by the ImageJ software (NIH, USA).

### Proteomic identification by mass spectrometry (MS) and data analysis

We assessed the expression of 3231 known proteins by performing mass spectrometry (MS) in human purified CD4 + T cells from healthy individuals (*n* = 3), RA untreated patients (*n* = 3), and RA patients treated with IL-6 receptor blockade (*n* = 3). All RA untreated patients are defined as naïve to treatment with disease-modifying anti-rheumatic and biological drugs and were seropositive for both rheumatoid factor and anti-citrullinated protein antibody. For proteomic analysis, MACS- purified CD4^+^ T cells were lysed in SP3 lysis buffer and chromatin was degraded with a Bioruptor. Samples were reduced with 5 mM Dithiothreitol (DTT) at 55 °C for 30 min, alkylated with 40 mM Chloroacetamide (CAA) at room temperature for 30 min and protein amount was quantified using the Direct Detect Spectrometer. The mass spectrometer was operated at CECAD/ZMMK Proteomics Facility (Cologne, Germany). LFQ values were log2 transformed. *T* test was used to determine significantly changing protein levels. *P* value of less than 0.05 as well as fold change of more than 1.5 was defined as significant differential expressed proteins (DFPs). Heat map visualization of DFPs was obtained calculating a z-score of the LFQ values for each protein by TBtools as described before [31].

### Gene ontology (GO), KEGG pathway analysis, and Gene set enrichment analysis (GSEA)

The top significantly expressed proteins (q less than 0.05) were subjected to GO, KEGG pathway analysis. STRING v1022 (http://string-db.org/) was used for data input, Gene Ontology, and pathway analysis. GO and KEGG analyzed data were visualized on a website: Weishengxin (http://www.bioinformatics.com.cn/). Gene set enrichment was performed using GSEA 4.0 software (http://www.gsea-msigdb.org/gsea/).

### P-VASP blocking antibody lipofection

Human purified CD4^+^ T cells were transfected by lipofection method using Pierce Protein Transfection Reagent Kit (Thermo Fisher Scientific, Massachusetts, USA) and P-VASP blocking peptide (Lifespan Biosciences Inc., Seattle, USA) reagent as recommended by the manufacturers. Briefly, the blocking peptide for P-VASP was diluted in DPBS (Gibco^®^ DPBS, New York, USA) before adding to the dried Pierce Reagent. Pipette up and down 3–5 times before vortex. After incubation at room temperature for 5 min, the lipo-surrounding P-VASP blocking peptide would be ready for subsequent experiments. The primary human CD4^+^ T cells were transfected by lipid surrounded P-VASP blocking antibody (Novus Biologicals, Centennial, USA) with the antibodies from T cell Activation/Expansion Kit (Miltenyi Biotec, Bergisch Gladbach, Germany) for an overnight incubation in a condition of 5% CO_2_ at 37 degree.

### Cell transwell migration assays

The migration of Treg cells was evaluated by chemo-attractant transwell migration assay. 6.5 mm Transwells with 5 µm pore size (Corning™ Incorporated Costar, New York, USA) were equilibrated for 2 h in x-vivo 15 media (Lonza, Cologne, Germany) supplied with 1% human serum and 1% penicillin–streptomycin (both Sigma-Aldrich, Saint Louis, USA). 1 × 10^6^ CD4^+^ T cells, were seeded to transwell and incubated for 4 h under cell culture condition. 50 ng/ml CCL20 (BioLegend Inc., San Diego, USA) was used as a chemo-attractant in the lower compartment. Total migrated CD4^+^ cells in the lower chamber were counted by hemocytometer and Treg cells were identified using anti-human CD25, CD127 and FoxP3 antibodies (all BioLegend Inc., San Diego, USA).

### Enzyme-linked immunosorbent assay (ELISA)

Mice serum preparation and IL-6 level in serum was performed according to the manufacturer’s instructions (Invitrogen, ThermoFisher Scientific, Carlsbad, USA).

### Statistics

Statistical analysis was performed using GraphPad Prism 8 (GraphPad Software, San Diego, USA). Data are presented as the mean ± SEM. Unpaired two-tailed Student’s t test or one-way ANOVA was used as appropriate. *p* < 0.05 was considered as statistically significant. *p < 0.05, **p < 0.01, ***p < 0.001, ns: non-significant, *p* > 0.05.

## Results

**Fig. 1:**
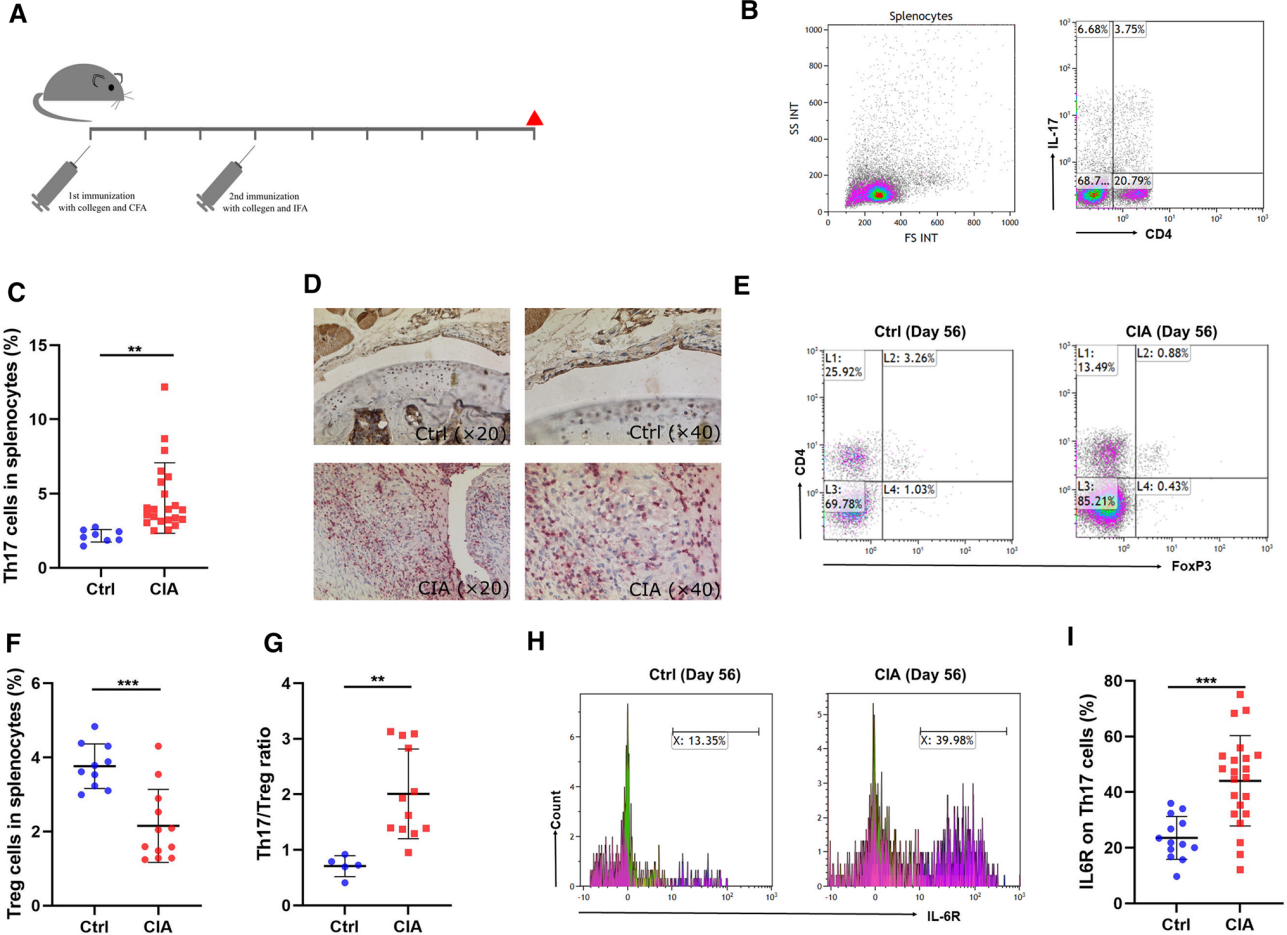
IL-6 receptor expression on Th17 cells is increased in experimental autoimmune arthritis. **a** Experimental setup. **b** Representative example of flow cytometry analysis of Th17 cells from the spleen of mice with collagen type II induced arthritis (CIA). **c** Frequency of Th17 cells in purified murine CD4^+^ splenocytes in the control group (Ctrl, *n* = 8) and the CIA group (*n* = 22) as assessed by flow cytometry. **d** Detection of Th17 cells by immunohistochemistry in the joints of murine hindlimbs (HLs) in control mice (Ctrl) and CIA mice. **e** Representative example of flow cytometry analysis of Treg cell frequency within murine CD4^+^ T cells sorted from splenocytes. **f** Treg cells frequencies analyzed by flow cytometry in purified murine CD4^+^ T cells isolated from splenocytes from control mice (n = 10) and CIA mice (*n* = 12). **g** Th17/Treg cell ratio in purified murine CD4^+^ splenocytes (Ctrl, *n* = 5; CIA, *n* = 12). **h** Representative example of flow cytometry analysis of membrane-bound IL-6 receptor on Th17 cells at day 56. **i** IL-6 receptor expression on Th17 cells in purified murine CD4^+^ splenocytes as assessed by flow cytometry (Ctrl, *n* = 13; CIA, *n* = 22). Data are presented as the mean ± standard error of the mean (SEM). Statistical analysis was performed using a two-tailed Student’s t test (*p < 0.05, **p < 0.01, ***p < 0.001)

We investigated dynamic characteristics in membrane-bound IL-6 receptor expression on Th17 cells in the CIA mouse model of autoimmune arthritis. A schematic experiment workflow is shown in Fig. 1a. No joint inflammation was observed in the control group which received PBS injections. A representative example of flow cytometry analysis of Th17 cells obtained from the spleen is shown in Fig. 1b. Our study confirms that Th17 cell frequencies are significantly increased in CIA mice as compared to control mice (Fig. 1c). In addition, immunohistochemistry staining of inflamed joints shows that Th17 cells migrate into the synovial tissue of CIA joints whereas no Th17 cells are found in the joint of control mice (Fig. 1d). In a next step, we analyzed the frequency of Treg cells in the spleen of mice with autoimmune arthritis. Figure 1e shows a representative example of flow cytometry analysis of Treg cells. In mice with CIA, Treg cell frequencies are significantly reduced as compared to control mice (Fig. 1f). As a consequence, the balance between Th17 cells and Treg cells is shifted towards Th17 cells in autoimmune arthritis (Fig. 1g). To further characterize Th17 cells, we analyzed the expression levels of membrane-bound IL-6 receptor by flow cytometry and found a significant increase in IL-6 receptor expression on Th17 cells in the CIA group (Fig. 1h, i).

### Dynamic upregulation of IL-6 receptor on Th17 cells is inversely correlated with IL-6 serum levels in autoimmune arthritis

**Fig. 2:**
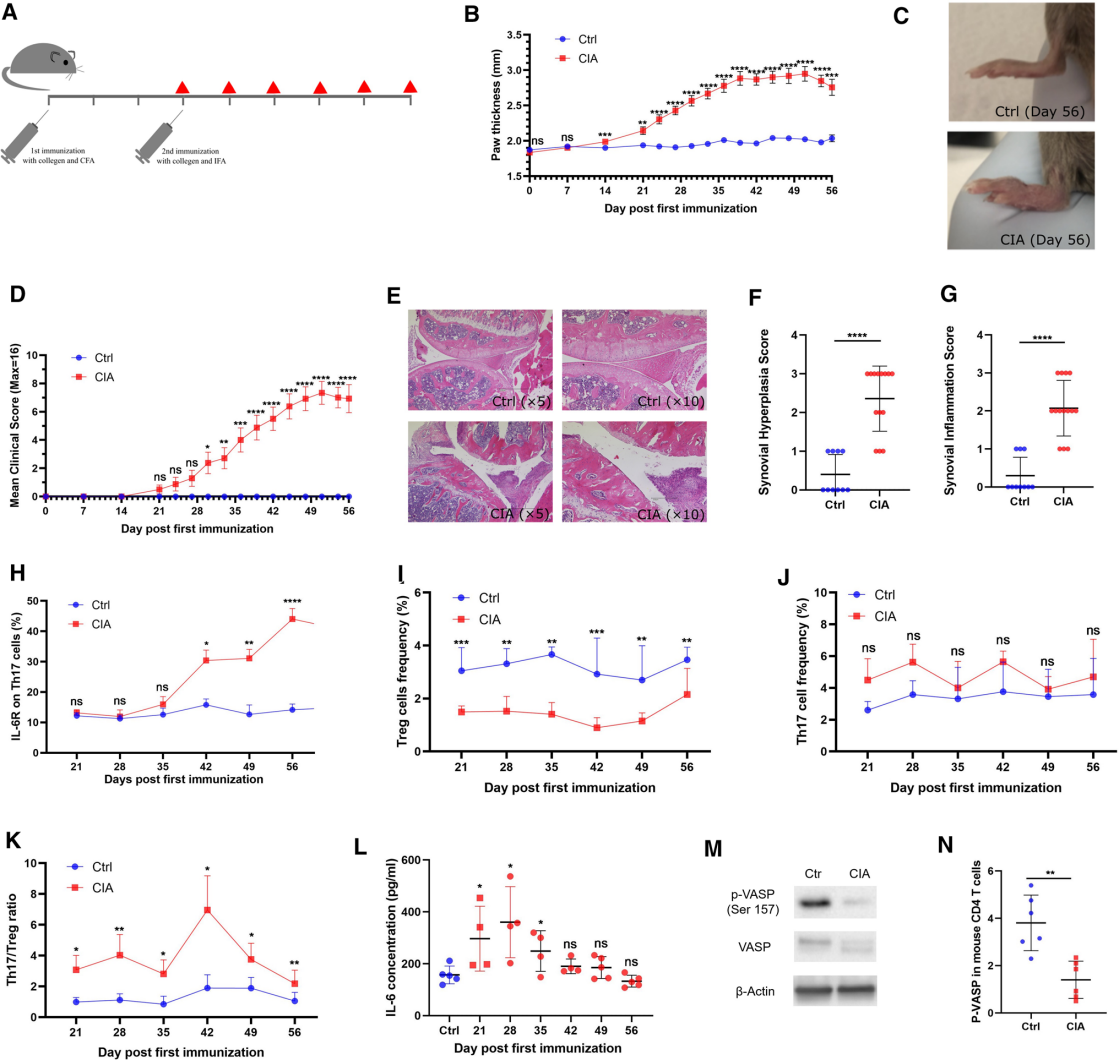
Dynamic IL-6 receptor upregulation on Th17 cells in experimental autoimmune arthritis. **a** Experimental setup. The red triangle represents the time point when mice were sacrificed. **b** Thickness of hindlimbs of control mice (*n* = 16) and CIA mice (*n* = 24). **c** Representative paws in CIA mice on day 56. **d** Mean clinical score of control mice (*n* = 16) and CIA mice (*n* = 24). **e** Representative example of hematoxylin and erosion (H&E) staining of hindlimbs from control mice and CIA mice. **f**, **g** Synovial hyperplasia and synovial inflammation in control mice (*n* = 10) and CIA mice (*n* = 14). **h** Flow cytometry analysis of membrane-bound IL-6 receptor expression on Th17 cells from control mice and CIA mice (*n* = 5 each). **i**, **j** Flow cytometry analysis of Treg cell frequency and Th17 cell frequency in CD4^+^ T cells isolated at different time points from splenocytes of control mice and CIA mice (*n* = 5 each). **k** Th17/Treg cell ratio at different time points during CIA development (*n* = 5 each). **l** IL-6 serum levels at different time points assessed by ELISA (*n* = 4/5 per group). **m** Representative example of western blot analysis of p-VASP expression in CD4^+^ T cells. **n** p-VASP (Ser157) expression in CD4^+^ T cells isolated from splenocytes of control mice and CIA mice (*n* = 6 per group). Data are presented as the mean ± standard error of the mean (SEM). Statistical analysis was performed using a two-tailed Student’s *t* test (*p < 0.05, **p < 0.01, ***p < 0.001, ****p < 0.0001)

After observing increased membrane-bound IL-6 receptor levels on Th17 cells from CIA mice, we aimed to study the dynamics of IL-6 receptor upregulation during the course of disease. We therefore monitored the expression levels of membrane-bound IL-6 receptor on Th17 cells in CIA. Mice were sacrificed at weekly intervals starting on the third week after the first immunization and the expression of IL-6 receptor on the cell surface of Th17 cells obtained from the spleen was analyzed by flow cytometry. The experimental setup is shown in Fig. 2a. The thickness of the paws of immunized mice started to grow significantly compared to healthy controls starting at day 21 after first immunization (Fig. 2b). A representative example of a swollen paw is shown in Fig. 2c. Furthermore, the mean clinical score of CIA mice was increased in a time-dependent manner (Fig. 2d). In addition, our histopathological analysis of the joints demonstrates a significantly higher synovial inflammation and increased synovial hyperplasia score in CIA mice in comparison to the control group (Fig. 2e–g). Monitoring of IL-6 receptor expression revealed a significant increase in membrane-bound IL-6 receptor levels on Th17 cells from CIA mice starting on day 42 after first immunization (Fig. 2h). High IL-6 receptor expression on Th17 cells was associated with a high clinical score in CIA mice. In contrast to IL-6 receptor expression on Th17 cells, Treg cell frequencies remain stable during the course of disease. However, the level of Treg cell frequencies is lower as compared to healthy controls and Treg cells from CIA mice express significantly less TGF-beta1 than Treg cells from control mice (Fig. 2i and Supplementary Fig. S1). Moreover, Treg cell frequencies and TGF-beta1 serum concentrations are diminished in transgenic mice overexpressing IL-6 (Supplementary Fig. S2 and S3). Th17 cell frequencies stay on a higher level, but no significant difference was found as compared to healthy controls (Fig. 2j). The ratio between Th17 cells and Treg cells is elevated in CIA mice as compared to healthy animals and shows a peak at day 42 after first immunization due to slightly increased Th17 cell frequencies and a mild reduction in Treg cell frequencies (Fig. 2k). However, no correlation with disease score is found. Remarkably, the IL-6 serum levels are inversely correlated with membrane-bound IL-6 receptor expression levels on Th17 cells and increase significantly during the first 28 days after first immunization, followed by a decrease starting on day 35 after first immunization (Fig. 2l). On day 56, the initial IL-6 serum levels are reached.

### Phosphorylation level of VASP is decreased in RA and restored by IL-6 receptor blockade

**Fig. 3:**
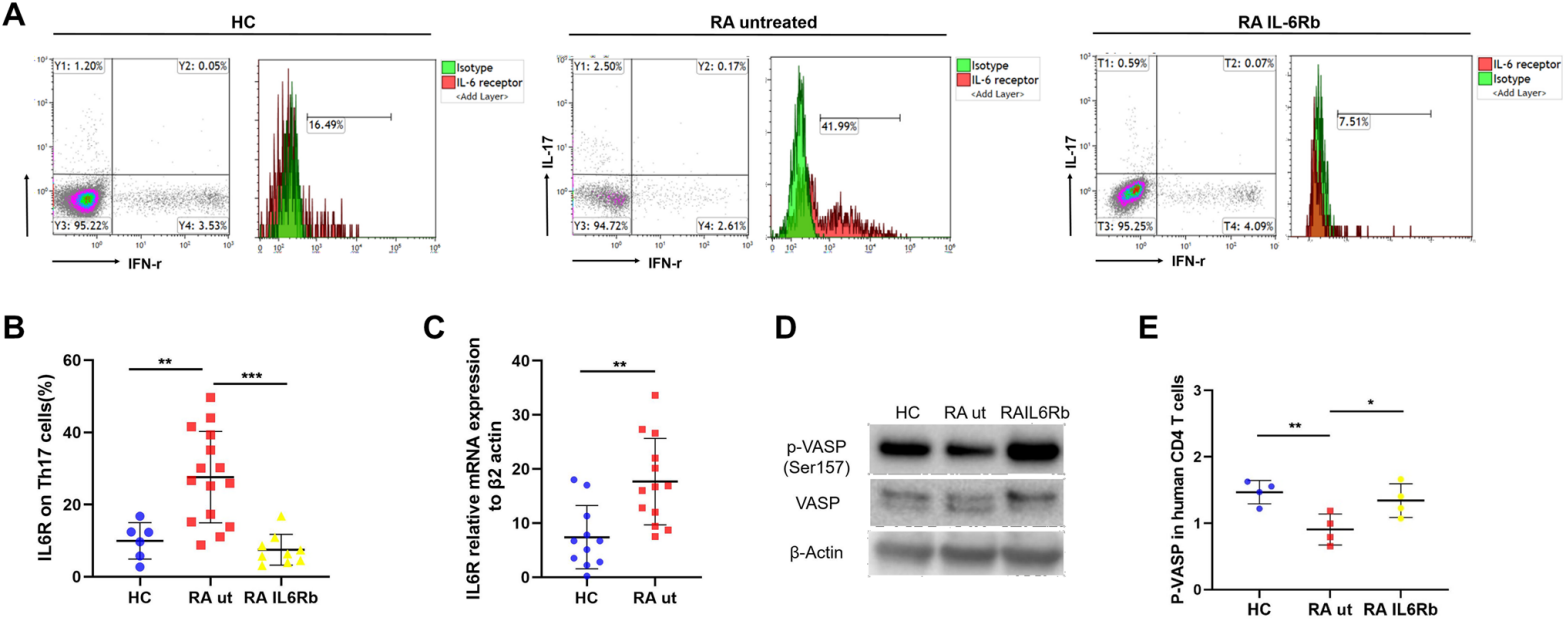
IL-6 receptor and p-VASP (Ser157) expression in RA patients. **a** Representative example of flow cytometry analysis of membrane-bound IL-6 receptor on Th17 cells in healthy controls (HC, left panel), untreated RA patients (panel in the middle) and RA patients treated with IL-6 receptor blocking antibodies (right panel). **b** Expression levels of membrane-bound IL-6 receptor on Th17 cells from healthy controls (HC, *n* = 6), untreated RA patients (RA ut, *n* = 15) and RA patients treated with IL-6 receptor blocking antibodies (RA IL6Rb, *n* = 8) as assessed by flow cytometry. **c** mRNA levels of IL-6 receptor (IL6R) relative to β-Actin in CD4^+^ T cells from HC (*n* = 11) and RA ut (*n* = 13). **d** Representative example of western blot analysis of p-VASP expression in CD4^+^ T cells. **e** p-VASP expression analysis by western blot in CD4^+^ T cells from HC (*n* = 4), RA ut (*n* = 6) and RA IL6Rb (*n* = 4). Data are presented as the mean ± standard error of the mean (SEM). Statistical analysis was performed using a two-tailed Student’s *t* test. One-way ANOVA analysis was performed for experiments with more than two groups. (*p < 0.05, **p < 0.01, ***p < 0.001)

IL-6 has been shown to reduce the phosphorylation of VASP, an important regulator of cell migration [19]. We therefore analyzed VASP phosphorylation in CD4^+^ T cells from CIA mice at day 56 after the first immunization. A representative western blot analysis of p-VASP (Ser 157) is shown in Fig. 2m. In CD4^+^ T cells from mice with CIA p-VASP is expressed at a significantly lower level as compared to control mice (Fig. 2n). Furthermore, p-VASP levels negatively correlate with the expression of IL-6R on Th17 cells and with the phosphorylation status of STAT3 (Supplementary Fig. S4 and S5). In a next step, we evaluated if the findings observed in CIA mice are also found in primary human cells from patients with RA. Peripheral blood mononuclear cells (PBMCs) were isolated from the peripheral blood of RA patients and healthy individuals, and CD4^+^ T cells were purified by MACS. Figure 3a shows a representative example of flow cytometry analysis of membrane-bound IL-6 receptor expression on Th17 cells from healthy controls, untreated RA patients and RA patients treated with the IL-6 receptor blocking antibody tocilizumab. Membrane-bound IL-6 receptor expression on Th17 cells is significantly upregulated in untreated RA patients (Fig. 3b). Interestingly, treatment with IL-6 receptor blocking antibodies inhibits IL-6 receptor expression as well as STAT3 phosphorylation and increases the frequency of Treg cells in the peripheral blood of RA patients (Fig. 3b and Supplementary Fig. S6, S7). In addition, IL-6 receptor expression is elevated on mRNA level in Th17 cells from untreated RA patients as compared to healthy controls (Fig. 3c). Consistent with western blots of p-VASP in CD4^+^ T cells from CIA mice, p-VASP (Ser 157) expression is significantly downregulated in untreated RA patients (Fig. 3d). Treatment with IL-6 receptor blocking antibodies restores the phosphorylation level of VASP in patients with RA (Fig. 3e).

### p-VASP expression level is associated with distinct protein expression profile in RA

**Fig. 4:**
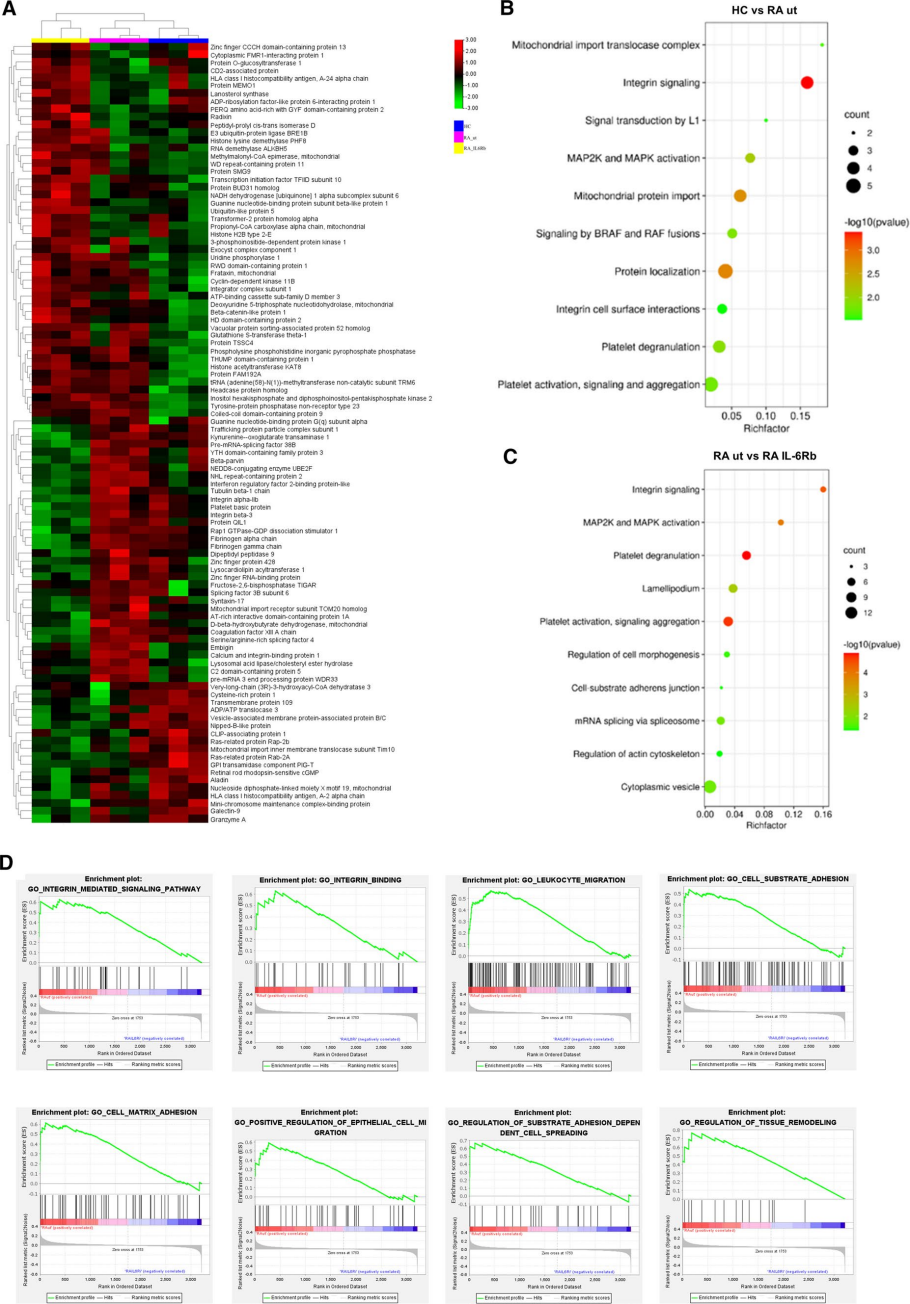
Proteomics analysis based on p-VASP (Ser157) levels in CD4^+^ T cells. **a** Heatmap of the relative abundances of proteins in three different types of samples (HC, blue columns; RA ut, pink columns; RA IL6Rb, yellow columns) (*n* = 3 per group). The expression patterns of the top 101 differentially expressed proteins are shown. Colored bars indicate the expression levels. Red blocks represent overexpressed proteins, blue blocks represent proteins with the lowest expression levels. **b**, **c** Bubble plots enriched on GO and KEGG pathway in CD4^+^ T cells from HC and RA ut (*n* = 3 per group), as well as RA ut and RA IL6Rb (*n* = 3 per group). **d** Representative enrichment plots are shown for categories identified using GSEA as significantly enriched in positively correlated proteins in RA ut as compared to RA IL6Rb. Integrin-mediated signaling pathway (*q* = 0.0016313214, NES: 1.708097), integrin binding (*q* = 0.012345679, NES: 1.6539156), leukocyte migration (*q* = 0.006825, NES: 1.913600), cell-substrate adhesion (*q* = 0.0, NES: 1.714524), cell matrix adhesion (*q* = 0.019293, NES: 1.838242), positive regulation of epithelial cell migration (*q* = 0.011308562, NES: 1.3389298), regulation of substrate adhesion-dependent cell spreading (*q* = 0.006102565, NES: 107215812), regulation of tissue remodeling (*q* = 0.0083289262, NES: 1.7024468). *NES* normalized enrichment score; *q* = FDR (false discovery rate) *q* value. Data are presented as the mean ± standard error of the mean (SEM). Statistical analysis was performed using a two-tailed Student’s *t* test. *p* < 0.05 was defined as significant. Heat map visualization of DFPs was obtained calculating a z-score of the label-free quantification values for each protein by TBtools. Bubble diagram was created on the website: http://www.bioinformatics.com.cn/. Gene set enrichment was performed using GSEA 4.0 software

Our next aim was to investigate possible links between the phosphorylation level of VASP and altered pathways in CD4^+^ T cells from RA patients. We therefore performed proteomic analysis of CD4^+^ T cells from RA patients with low p-VASP expression and compared the results to CD4^+^ T cells from healthy individuals with high p-VASP level. We found that 33 proteins are upregulated and 26 proteins are downregulated (more than 1.5 fold change) in cells with high p-VASP levels (Fig. 4a). These proteins are enriched in various pathways including but not limited to integrin signaling, signal transduction by L1, MAP2K and MAPK activation, mitochondrial protein import, protein localization and integrin cell surface interactions (Fig. 4b). Compared to the group with low p-VASP expression group, 17 proteins are upregulated in RA patients treated with IL-6 receptor blocking antibodies (and high p-VASP expression) and 34 proteins are downregulated (more than 1.5 fold change) (Fig. 4a). These differentially expressed proteins (DEPs) are enriched in pathways including integrin signaling, MAP2K and MAPK activation, regulation of cell morphogenesis, cell-substrate adhesion junction, mRNA splicing via spliceosome, regulation of actin cytoskeleton, and cytoplasmic vesicle (Fig. 4c). The DEPs are involved in three functional categories, including biological processes, molecular functions and molecular components. Interestingly, DEPs that are involved in integrin signaling were upregulated in the group with low p-VASP expression but downregulated in RA patients with high p-VASP expression treated with IL-6 receptor blocking antibodies. Integrin signaling pathways therefore seem to be important pathways in RA and they are modified by treatment with IL-6 receptor blocking antibodies.

### Gene set enrichment analysis (GSEA) based on proteomics

In a next step, gene set enrichment analysis (GSEA) was performed based on DEPs. Several pathways closely related to CD4^+^ T cell functions are significantly modified in untreated RA patients with low p-VASP expression (Fig. 4d): integrin mediated signaling pathway (*q* = 0.0016313214, NES: 1.708097), integrin binding (*q* = 0.012345679, NES: 1.6539156), leukocyte migration (*q* = 0.006825, NES: 1.913600), cell-substrate adhesion (*q* = 0.0, NES: 1.714524), cell matrix adhesion (*q* = 0.019293, NES: 1.838242), positive regulation of epithelial cell migration (*q* = 0.011308562, NES: 1.3389298), regulation of substrate adhesion-dependent cell spreading (*q* = 0.006102565, NES: 107215812), regulation of tissue remodeling (*q* = 0.0083289262, NES: 1.7024468).

### Reduced migration of Treg cells, but not effector CD4^+^ T cells, following p-VASP specific blockade

**Fig. 5:**
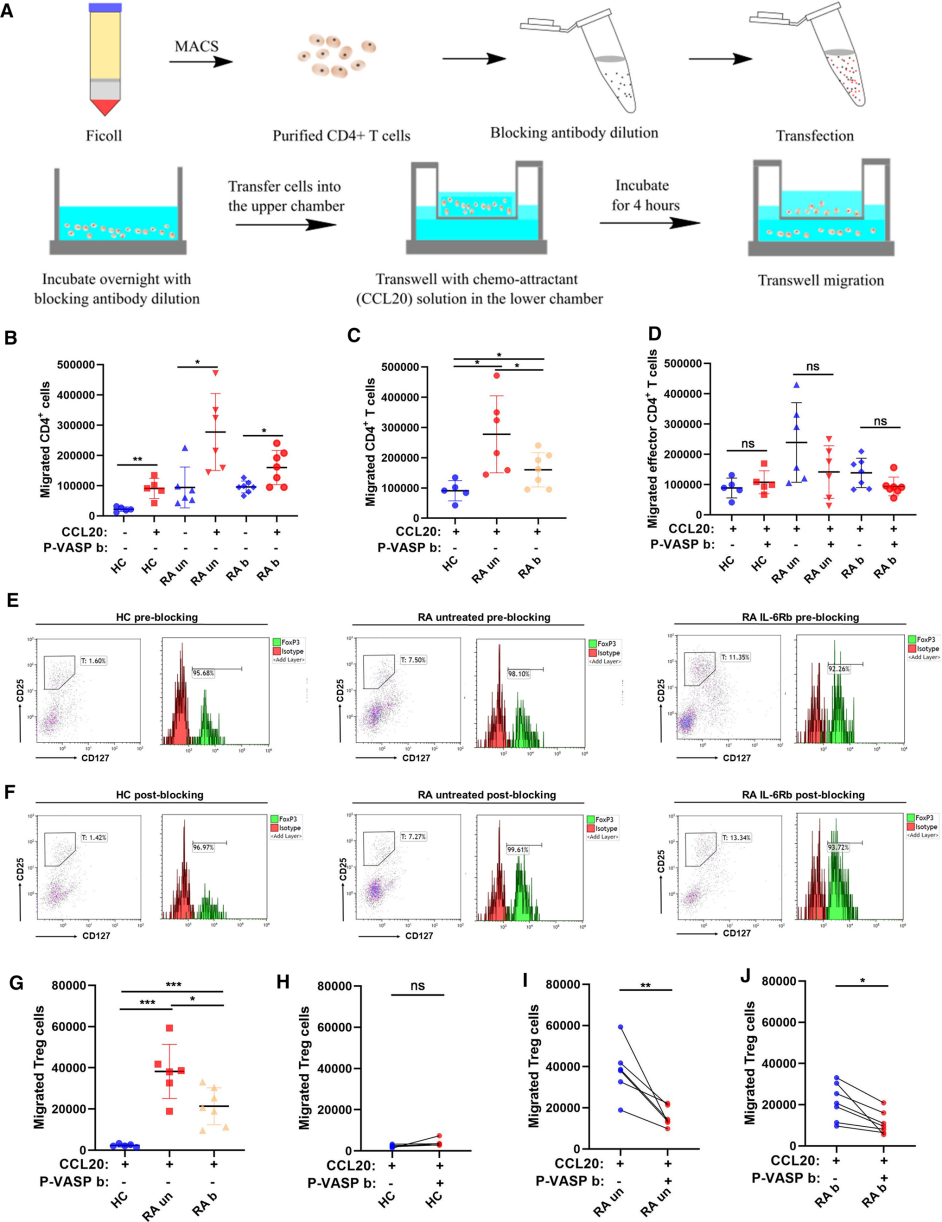
p-VASP is implicated in Treg cell migration, but not in effector T cell migration. **a** Schematic view of the experimental setup. **b** Absolute number of migrated *total* CD4^+^ T cells (including Treg cells) in the negative and positive control group in a chemo-attractant transwell system. **c** Absolute number of migrated *total* CD4^+^ T cells (including Treg cells). For clarity, the data from Fig. 5B are presented in a different way to show the differences between HC, RA un and RA b in the CCL20 group. **d** Absolute number of migrated *effector* CD4^+^ T cells (without Treg cells) after specific blockade. **e**,** f** Representative flow cytometry plots showing migrated Treg cells in percentage without and with p-VASP blocking antibodies. **g** Absolute number of migrated Treg cells by flow cytometry. **h-j** Migrated Treg cells from HC, RA un, and RA b without and with p-VASP specific blocking antibodies. The data without p-VASP blocking antibodies are the same as shown in (**g**). For clarity, they are presented for comparison to p-VASP blocking antibodies in (**h-j**). (HC, healthy controls, *n* = 5; RA un, untreated RA patients, *n* = 6; RA b, RA patients treated with IL-6 receptor blocking antibodies, *n* = 7). Data are presented as the mean ± standard error of the mean (SEM). Statistical analysis was performed using a two-tailed Student’s *t* test. One-way ANOVA analysis is performed in more than two groups (*p < 0.05, **p < 0.01, ***p < 0.001)

To further identify whether p-VASP (Ser 157) is crucial for the migration of immune cells in RA, we specifically blocked it and performed transwell migration assay with purified CD4^+^ T cells from RA patients as well as healthy individuals in vitro. The chemo-attractant migration assay was performed following the workflow shown in Fig. 5a. A chemo-attractant transwell assay experiment (50 ng/ml CCL20 in the lower chamber) was performed to study migration of T cells. More CD4^+^ T cells migrated towards media containing CCL20 than to control media without CCL20 (Fig. 5b). The experiment revealed that CD4^+^ T cell migration is increased in untreated RA patients whereas it is reduced in RA patients treated with IL-6 receptor blocking antibodies (Fig. 5c). Regarding the migration of effector CD4^+^ T cells, no significant difference was found before and after specifically blocking p-VASP (Ser 157) in healthy individuals, untreated RA patients, and RA patients treated with IL-6 receptor blockade (Fig. 5d). We therefore analyzed the influence of p-VASP on Treg cell migration. Representative examples of flow cytometry analysis of migrated CD25^high^CD127^low^FoxP3^+^ Treg cells from healthy individuals, untreated RA patients and RA patients treated with IL-6 receptor blocking antibodies with or without specific p-VASP blocking antibodies are displayed in Fig. 5e, f, respectively. In RA patients, more migrated Treg cells were found as compared to healthy individuals (Fig. 5g). In healthy individuals, no difference before and after blocking of p-VASP can be observed (Fig. 5h). In contrast, the absolute number of migrated Treg cells decreases after specific blockade of p-VASP (Ser 157) in untreated RA patients and RA patients treated with anti-IL-6 receptor antibodies (Fig. 5i, j). These results show that p-VASP is implicated in Treg cell migration in RA.

## Discussion

It is proved that IL-6 regulates the proliferation of Th17 cells through the JAK/STAT3 signaling pathway [25, 32, 33]. Recently, elevated IL-6 receptor expression on CD4^+^ T cells was reported to promote Th17 cell-driven inflammation by increasing IL-17 production in autoimmune diseases [34]. It has been shown that increased IL-6 receptor expression on CD4^+^ T cells can be induced by T cell activation via TCR engagement [35, 36]. Here we describe a dynamic upregulation of IL-6 receptor expression during the course of experimental autoimmune arthritis which is negatively correlated with IL-6 serum levels. This upregulation could be an important mechanism contributing to the maintenance of Th17 cell functions in autoimmune arthritis when IL-6 serum level decreases. Consistent with our results obtained from animal studies, our flow cytometry and qRT-PCR analysis of human IL-6 receptor expression on Th17 cells revealed a significant increase in IL-6 receptor expression in untreated RA patients. In a recent study, Harbour et al. [18] describe that persistent IL-6 signaling through ongoing classical IL-6 receptor activation is required to retain the transcriptional and functional identity of Th17 cells in two mouse models of colitis. In contrast, Nowell et al*.* report that IL-6 receptor is rapidly downregulated on human leukocytes from the peripheral blood and the synovial fluid after activation of the T cell receptor [37]. These contradictory results can be explained by the fact that Nowell et al*.* studied the influence of T cell receptor signaling on IL-6 receptor expression in leukocytes, while IL-6 receptor expression on Th17 cells may be regulated differently as compared to other CD4^+^ T cell subsets. Our results provide evidence of a possible link between IL-6 serum levels and IL-6 receptor expression on Th17 cells, thereby suggesting that IL-6 receptor upregulation is a pathogenic mechanism contributing to the maintenance of pathogenic Th17 cell functions in autoimmune arthritis.

Post-translational modification of VASP by phosphorylation is an important regulatory mechanism in cell migration of fibroblasts and cancer cells [20–22]. Moreover, Hebatullah et al*.* reported that VASP is a major regulator of leukocyte recruitment in post-ischemic revascularization [38]. In addition, Henes et al*.* found a reduced level of VASP in endothelial HMEC-1 cells in response to IL-6 [19]. Considering the role of p-VASP in cell migration and the influence of IL-6 on p-VASP levels in our experiments, we hypothesized that IL-6 may regulate Treg cell migration through post-translational modification of VASP. To verify our hypothesis, we performed T cell migration assays and specifically blocked p-VASP (Ser 157), which is downregulated in murine CD4^+^ T cells from CIA mice and in CD4^+^ T cells from untreated RA patients. The results of our experiments confirmed a specific role of p-VASP in Treg cell migration in RA, but not in Treg cells from healthy individuals.

We performed proteomics based on purified CD4^+^ T cells from healthy individuals, untreated RA patients and RA patients treated with IL-6 receptor blocking antibodies to identify pathways that might be affected by changes in phosphorylation of VASP. Figure 4a shows three groups of DEPs. In a next step, gene ontology (GO) and KEGG pathway analysis based on DEPs were performed. Our results highlight the role of integrin signaling as the activation of related pathways were significantly enriched in the group with reduced expression of p-VASP (Ser 157). Furthermore, the reversibility of reduced p-VASP expression in RA patients by IL-6 receptor inhibition confirms a possible link between IL-6 and p-VASP. GSEA confirmed the enrichment of integrin signaling and related pathways in CD4^+^ T cells with low p-VASP (Ser 157) levels (Fig. 4d). Consistent with our results, previous evidence suggest that integrin signaling implies cell adhesion to the extracellular matrix and to other cells [39–41]. Collectively, these data indicate p-VASP may play a role in CD4^+^ T cell migration in autoimmune arthritis. Therefore, we evaluated whether p-VASP (Ser 157) regulates the migration of Treg cells. To confirm this hypothesis, we specifically blocked p-VASP in purified human CD4^+^ T cells in vitro and performed flow cytometry analysis of migrated CD4^+^ effector T cells and Treg cells. Our study shows that specific blockade of p-VASP has no influence on CD4^+^ effector T cell migration. In contrast, Treg cell migration is significantly reduced by specific inhibition of p-VASP (Ser 157) with blocking antibodies in untreated RA patients as well as in RA patients treated with IL-6 receptor blocking antibodies. The observed increase in Treg cell migration in RA explains why Treg cell frequencies are elevated in the synovial fluid of RA patients in some studies [14, 42, 43]. In contrast to Treg cells from RA patients, absolutely no reduction in cell migration is seen in Treg cells from healthy individuals following specific blockade of VASP. In accordance with previous studies on CD4^+^ T cells in RA [44–46], our migration assay shows that the total number of migrated CD4^+^ T cells is significantly higher in untreated RA patients. This effect seems to exceed the relative increase in Treg cell migration in RA patients compared to healthy individuals.

One of the limitations of this study is that the functional role of VASP for Treg cell migration is not studied in VASP knock-out mice. This approach is hampered by the fact that knock-out of VASP affects the cortical development and leads to the absence of major cortical axon tracs. VASP knock-out mice therefore die during embryogenesis [47]. Despite the lack of in vivo data, we believe that the results of our ex vivo and in vitro analysis of murine and human CD4^+^ T cells strongly support a role of VASP in Treg cell migration.

To summarize, our current study provides insight into the characteristics of dynamic increase of IL6 receptor expression on Th17 cells and links IL-6 receptor signaling to reduced migration of Treg cells. Modification of Treg cell migration is induced by IL-6-mediated reduction in post-translational phosphorylation of VASP. Our findings identify impaired VASP phosphorylation as an important process contributing to deficient Treg cell migration and thereby to the pathogenesis of RA. Modification of VASP phosphorylation may be a promising therapeutic strategy to ameliorate autoimmune arthritis by enhanced Treg cell migration into inflamed joints.

## Supplementary Information

Below is the link to the electronic supplementary material.

Supplementary file1 (DOCX 1296 KB)

## Data Availability

The datasets generated during the current study are available from the corresponding author on reasonable request.

## Abbreviations

CFA: Complete Freund’s adjuvant
CIA: Collagen-induced arthritis
DAS28: Disease activity score (determined by 28 joint count)
DEPs: Differential expressed proteins
ELISA: Enzyme-linked immunosorbent assay
GO: Gene ontology
GSEA: Gene set enrichment analysis
H&E: Hematoxylin and eosin (H&E)
IFA: Incomplete Freund’s adjuvant
KEGG: Kyoto Encyclopedia of Genes and Genomes
MACS: Magnetic-activated cell sorting
MS: Mass spectrometry
TCR: T cell receptor
NES: Normalized enrichment score
PBMC: Peripheral blood mononuclear cells
qPCR: Quantitative real-time PCR
RA: Rheumatoid arthritis
Treg: Regulatory T cells
VASP: Vasodilator-stimulated phosphoprotein
WB: Western blot
