# Supplementary material for: Membrane-bound IL-6R is upregulated on Th17 cells and inhibits Treg cell migration by regulating post-translational modification of VASP in autoimmune arthritis
Source: Cell Mol Life Sci. 2021 Dec 16;79(1):3. doi: 10.1007/s00018-021-04076-2 (PMC8674172; doi:10.1007/s00018-021-04076-2)
Supplement: Supplementary file 1 — Supplementary file1 (DOCX 1296 KB) [file 18_2021_4076_MOESM1_ESM.docx]

**Supplementary Information**

Supplementary Figures S1 – S8

Membrane-bound IL-6R is upregulated on Th17 cells and inhibits Treg cell migration by regulating post-translational modification of VASP in autoimmune arthritis

Yan et *al.*


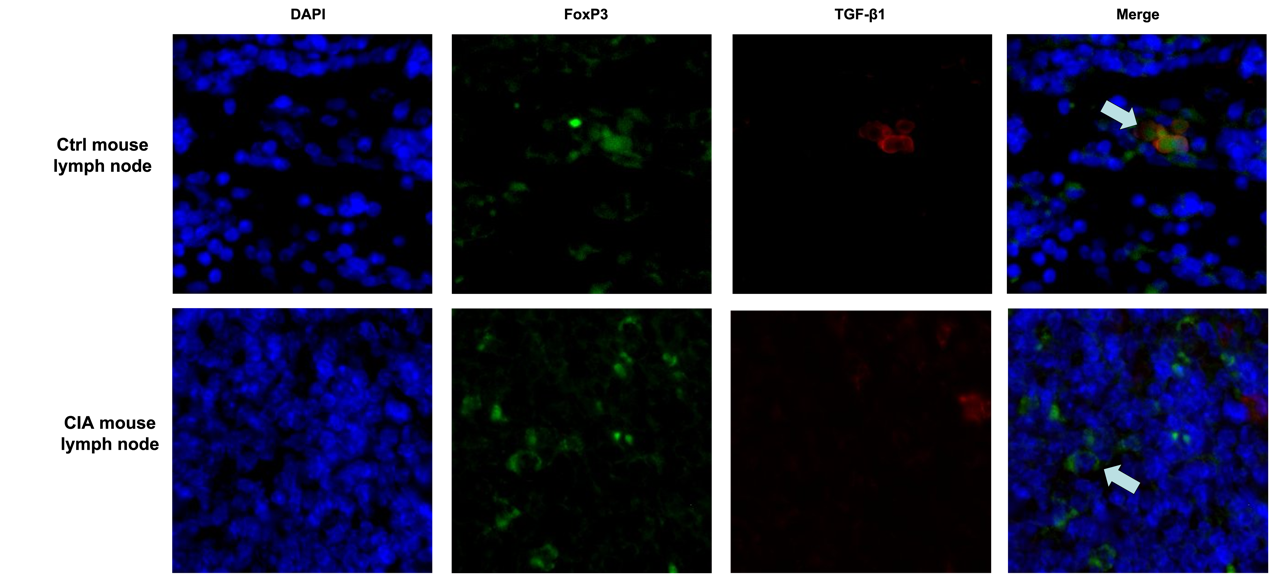


**Suppl. Fig. S1.** Representative immunofluorescence (IF) staining of TGF-beta1 and FoxP3 in lymph nodes from control mice (upper raw) and CIA mice (lower raw). Treg cells are marked with an arrow.


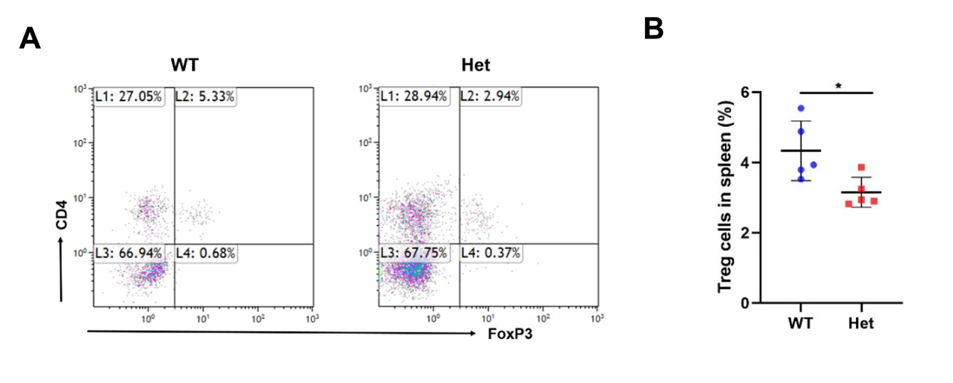


**Suppl. Fig. S2.** Treg cells frequency in wide type (WT) and heterozygous (Het) mouse that overexpresses cytokine IL-6. (**A**) Representative example of flow cytometry staining (**B**) Treg cell frequencies in the spleen. Data are presented as the mean ± standard error of the mean (SEM). Statistical analysis was performed using a two-tailed Student’s t-test (**p* < 0.05).


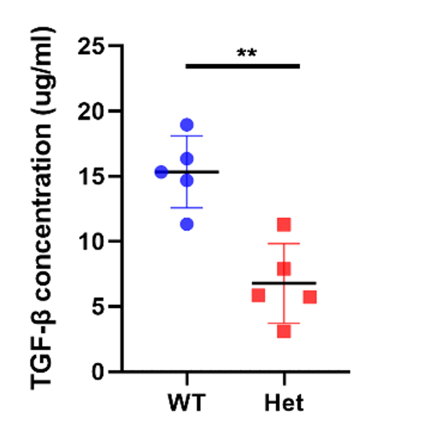


**Suppl. Fig. S3.** TGF-beta1 in mouse serum from wide type (WT) mice and heterozygous IL-6 overexpressing (Het) mice. Data are presented as the mean ± standard error of the mean (SEM). Statistical analysis was performed using a two-tailed Student’s t-test (***p* < 0.01).


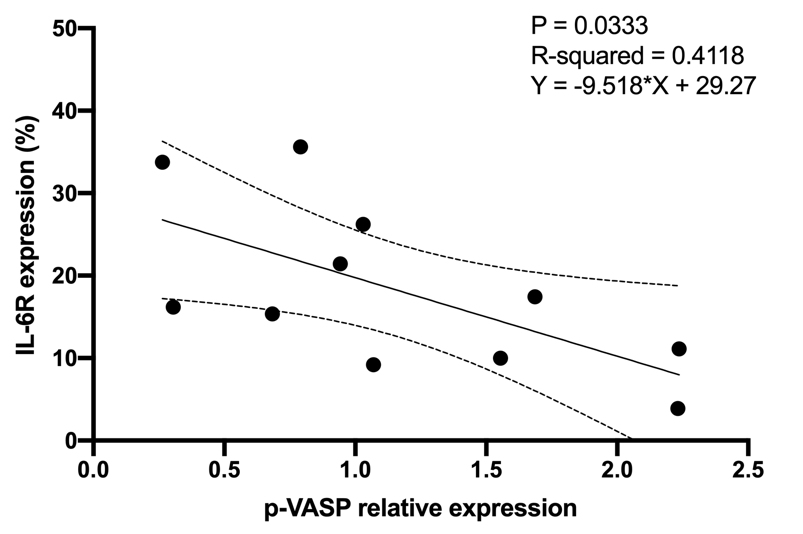


**Suppl. Fig. S4.** Correlation between L-6R expression on Th17 cells and p-VASP expression. IL-6R was analyzed by flow cytometry and p-VASP by western blot. (*p*=0.0333, R^2^=0.4118, Y=-9.518*X+29.27). The statistical analysis was performed by Spearman r correlation test (n=11).


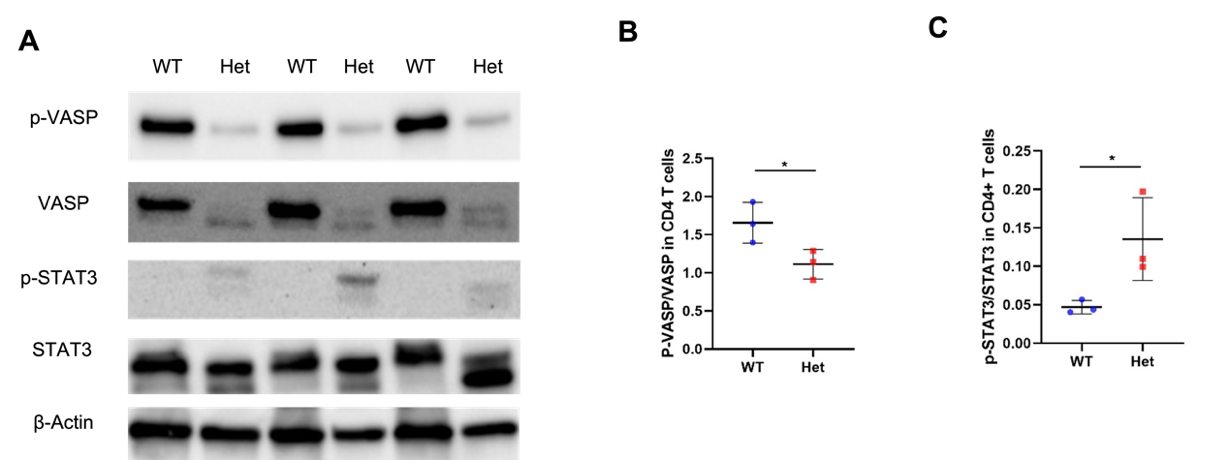


**Suppl. Fig. S5.** Western blot analysis of protein expression in CD4+ T cells from wide type (WT) mice and heterozygous (Het) mice that overexpresses IL-6. (**A**) Representative example of western blot analysis of STAT3, p-STAT3, VASP, and p-VASP in CD4+ T cells from WT and Het mice. (**B**) Relative expression of p-VASP and (**C**) p-STAT3 in CD4+ T cells from WT mice and Het mice. Data are presented as the mean ± standard error of the mean (SEM). Statistical analysis was performed using a two-tailed Student’s t-test (**p* < 0.05).

**
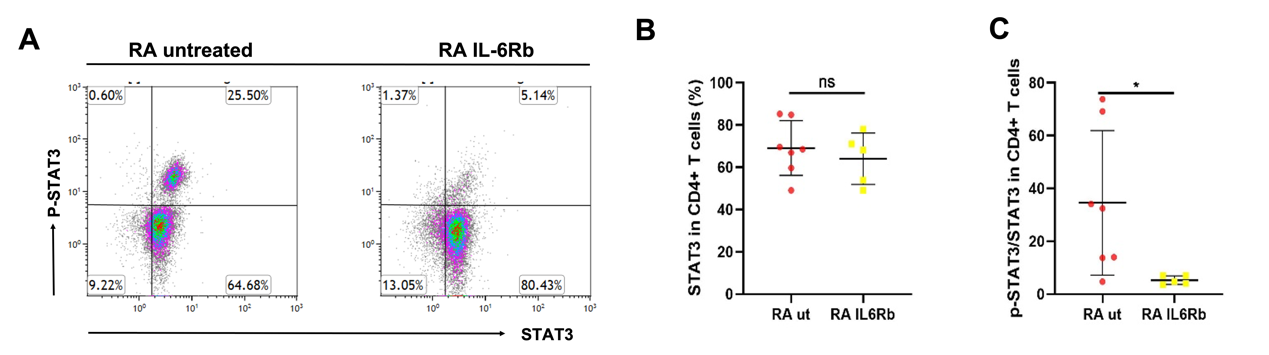
**

**Suppl. Fig. S6.** STAT3 phosphorylation level in CD4+ T cells from untreated RA patients (RA ut) and from patients treated with IL-6Rb (RA IL6Rb). (**A**) Representative example of flow cytometry staining of STAT3 and p-STAT3. (**B**) STAT3 and (**C**) p-STAT3 expression in CD4+ T cells. Data are presented as the mean ± standard error of the mean (SEM). Statistical analysis was performed using a two-tailed Student’s t-test (**p* < 0.05; ns = no significance).


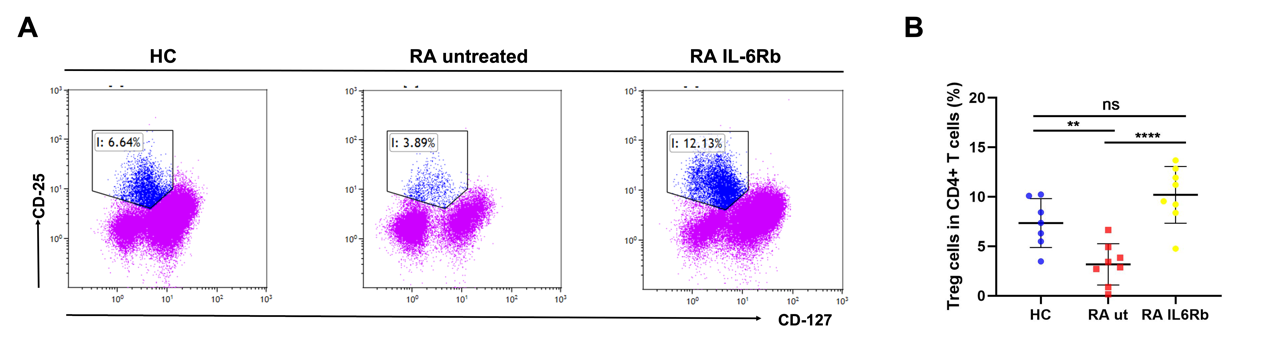


**Suppl. Fig. S7.** Treg cell frequencies in peripheral CD4+ T cells from healthy controls (HC), untreated RA patients (RA ut) and RA patients treated with IL-6R blocker (RA IL6Rb). (**A**) Representative example of flow cytometry analysis of CD25^high^CD127^low^ Treg cells. (**B**) Treg cell frequencies in peripheral CD4+ T cells. Data are presented as the mean ± standard error of the mean (SEM). Statistical analysis was performed using one-way ANOVA (**p* < 0.05, ***p* < 0.01, ****p* < 0.001, *****p* < 0.0001; ns = no significance).
